# Phase 1 study of telisotuzumab vedotin in Japanese patients with advanced solid tumors

**DOI:** 10.1002/cam4.3815

**Published:** 2021-03-06

**Authors:** Yutaka Fujiwara, Hirotsugu Kenmotsu, Noboru Yamamoto, Toshio Shimizu, Kan Yonemori, Christopher Ocampo, Apurvasena Parikh, Sumiko Okubo, Kazuteru Fukasawa, Haruyasu Murakami

**Affiliations:** ^1^ National Cancer Center Hospital Tokyo Japan; ^2^ Shizuoka Cancer Center Shizuoka Japan; ^3^ AbbVie Inc North Chicago IL USA; ^4^ AbbVie Inc Redwood City CA USA; ^5^ AbbVie GK Osaka Japan; ^6^ AbbVie GK Tokyo Japan

**Keywords:** Antibody‐drug conjugate, Cancer, Clinical trial, c‐Met, Japanese patients

## Abstract

Telisotuzumab vedotin (formerly ABBV‐399) is an antibody‐drug conjugate targeting c‐Met–overexpressing tumor cells, irrespective of *MET* gene amplification status. Safety, pharmacokinetics, and preliminary efficacy of telisotuzumab vedotin were evaluated outside of Japan. This phase 1 open‐label study evaluated the safety, tolerability, pharmacokinetics, and preliminary antitumor activity of telisotuzumab vedotin in Japanese patients with advanced solid tumors. Telisotuzumab vedotin was administered intravenously at either 2.4 mg/kg (n = 3) or 2.7 mg/kg (n = 6) every 3 weeks, following a 3 + 3 design. Maximum tolerated dose was not reached on the basis of the study design; no dose‐limiting toxicity events were observed. The most common treatment‐emergent adverse events related to telisotuzumab vedotin were peripheral sensory neuropathy (44%), and nausea, decreased appetite, and decreased white blood cell count (33% each). Most frequent grade ≥3 treatment‐emergent adverse events, irrespective of relationship to telisotuzumab vedotin, were decreased neutrophil count and hypoalbuminemia, reported in two patients (22%) each. Systemic exposure of telisotuzumab vedotin at both dose levels was approximately dose proportional. Pharmacokinetic profile in Japanese patients was similar to that previously reported in non‐Japanese patients. Two (22%) patients achieved a partial response, six (67%) had stable disease, one (11%) had progressive disease. Overall disease control rate was 89% (eight of nine patients; 95% confidence interval: 51.8%–99.7%]). Median progression‐free survival was 7.1 months (95% confidence interval: 1.2–10.4). In conclusion, telisotuzumab vedotin demonstrated a manageable safety profile, with antitumor activity in Japanese patients with advanced solid tumors; the recommended phase 2 dose was confirmed as 2.7 mg/kg every 3 weeks.

ClinicalTrials.gov registration number: NCT03311477.

## Graphical abstract

Telisotuzumab vedotin (formerly ABBV‐399) is an antibody‐drug conjugate targeting c‐Met–overexpressing tumor cells, irrespective of MET gene amplification status. This phase 1 open‐label study evaluated the safety, tolerability, pharmacokinetics, and preliminary antitumor activity of telisotuzumab vedotin in Japanese patients with advanced solid tumors. Telisotuzumab vedotin demonstrated a manageable safety profile, with antitumor activity in Japanese patients with advanced solid tumors; the recommended phase 2 dose was confirmed as 2.7 mg/kg every 3 weeks.

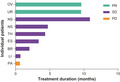

## 1 INTRODUCTION

c‐Met is a receptor tyrosine kinase expressed on the surface of epithelial and endothelial cells and is activated in various tumor types as a result of gain‐of‐function *MET* mutations, *MET* amplification, and c‐Met overexpression.^1^ Binding of the hepatocyte growth factor (HGF) ligand to c‐Met activates signaling pathways involved in cell survival, growth, migration, invasion, and metastasis.^2^, ^3^ Abnormal c‐Met activation is reported in many types of solid tumors, including non‐small cell lung cancer (NSCLC),^4^, ^5^ ovarian cancer,^6^, ^7^ breast cancer,^8^ prostate cancer,^9^ and others.^10^, ^11^

HGF binding of c‐Met has been shown to accelerate the development of *MET* genomic amplification in vitro and in vivo.^12^ Whereas primary *MET* amplification is a low‐frequency event that occurs in around 1% to 5% of tumor cell clones,^13^, ^14^ higher frequencies of *MET* amplification are found in patients with advanced and/or recurrent tumors.^5^, ^13^, ^15^ In patients with epidermal growth factor receptor (*EGFR*)‐mutated NSCLC who progress during treatment with EGFR inhibitors, *MET* amplification is detected in around 20% of cases.^16^, ^17^ Both *MET* amplification and c‐Met overexpression have been associated with poor clinical outcomes, underscoring the importance of increased c‐Met signaling in some cancer types.^5^, ^7^, ^11^, ^15^ Moreover, aberrant c‐Met signaling is associated with acquired resistance to EGFR inhibitors.^4^, ^5^ Collectively, these observations suggest a strong rationale for targeting c‐Met in patients whose tumors show aberrant c‐Met expression.

To date, there is a limited number of approved drugs in Japan that target the c‐Met pathway, including crizotinib and cabozantinib.^18^, ^19^ Many others are currently being evaluated in clinical trials, but despite encouraging activity in early phase studies, recent phase 3 trials have failed to show significant clinical benefit in patients with c‐Met–positive tumors.^20^, ^21^, ^22^ Another phase 3 trial of the c‐Met inhibitor tivantinib in Asian patients with previously treated *EGFR*‐wildtype NSCLC found potential antitumor activity, but the trial was prematurely terminated due to an increased incidence of interstitial lung disease in the tivantinib arm.^23^ One promising line of research is the use of spectrum‐selective c‐Met inhibitors, which may have potential as antitumor therapy in patients with *MET* exon 14 deletion.^24^ Encouraging results have recently been reported with kinase inhibitors targeting the c‐Met pathway in patients with *MET* exon 14‐mutated NSCLC.^25^, ^26^ However, there remains an unmet treatment need for novel c‐Met–targeting agents that can alter the natural course of disease in patients with advanced solid tumors and c‐Met protein overexpression.

Telisotuzumab vedotin (teliso‐v, formerly ABBV‐399) is a first‐in‐class antibody‐drug conjugate (ADC) composed of the anti–c‐Met humanized monoclonal antibody ABT‐700 coupled to cytotoxic monomethyl auristatin E (MMAE) through a valine‐citrulline linker with a drug:antibody ratio of approximately three.^27^ Teliso‐v targets c‐Met–overexpressing tumor cells, irrespective of *MET* gene amplification status, resulting in blockade of both HGF‐dependent and HGF‐independent c‐Met signaling. It is then internalized and releases the cytotoxin MMAE directly into the tumor cell, leading to tumor cell death.^28^ The first‐in‐human study of teliso‐v, conducted outside of Japan (NCT02099058), demonstrated its favorable safety and tolerability profiles, with encouraging evidence of antitumor activity in patients with c‐Met–positive NSCLC.^3^ However, clinical evaluation of teliso‐v in Japanese patients had not been carried out thus far.

Given the need for improved treatment options, we conducted a phase 1 study of teliso‐v in Japanese patients with advanced solid tumors, to evaluate the safety, tolerability, pharmacokinetic (PK), and preliminary antitumor activity of teliso‐v in this population.

## 2 METHODS

### 2.1 Study design

This phase 1, open‐label, dose‐escalation clinical trial was conducted at two sites in Japan (NCT03311477). Enrollment began on November 6, 2017, and the trial was completed on March 4, 2019. The primary objectives of the study were to evaluate the safety, tolerability, and PK of teliso‐v. The secondary objective was to assess the preliminary antitumor efficacy of teliso‐v.

Patients were enrolled at two teliso‐v dose levels, following a 3 + 3 design. Considering that 2.7 mg/kg every 3 weeks (Q3 W) was determined to be the recommended phase 2 dose (RP2D) in the preceding phase 1 study, the reported clinical study administered teliso‐v at dose levels from 2.4 mg/kg to 2.7 mg/kg. Escalation into the higher dose level proceeded if the first three evaluable patients in the lower dose level completed the first‐cycle (for at least 21 days) safety assessment without experiencing a dose‐limiting toxicity (DLT). DLT evaluations for dose escalation and determination of RP2D were carried out by investigators, study sponsor, and an independent safety monitor. Teliso‐v was administered intravenously over 30 minutes at either 2.4 or 2.7 mg/kg Q3 W on day 1 of a 21‐day cycle. No premedication was required. On the basis of confirmed availability of archival tumor tissue at enrollment, patients were retrospectively analyzed for c‐Met expression and *MET* amplification. c‐Met expression was determined by immunohistochemistry (IHC), and *MET* amplification was determined by fluorescence in situ hybridization or sequencing of plasma or tumor DNA.

All patients provided written informed consent, and the study was conducted in accordance with its protocol, International Conference on Harmonization Good Clinical Practice guidelines, applicable regulations and guidelines governing clinical study conduct, and ethical principles that have their origin in the Declaration of Helsinki. This study has been approved by the Independent Ethics Committee/Institutional Review Board of each participating site.

### 2.2 Patients

The trial enrolled Japanese patients (age ≥20 years) with histologically confirmed advanced solid tumors not amenable to surgical resection or other approved therapeutic options that have demonstrated clinical benefit. Other key inclusion criteria were Eastern Cooperative Oncology Group (ECOG) performance status 0–2, measurable disease per Response Evaluation Criteria In Solid Tumors (RECIST) version 1.1, availability of archived diagnostic formalin‐fixed paraffin embedded tumor tissue for analysis, and adequate bone marrow, renal, and hepatic function. Patients with local or central laboratory data showing the presence of c‐Met overexpression, *MET* exon 14 mutation, or *MET* amplification in the absence of archival tumor tissue availability remained eligible for enrollment at the sponsor's discretion. An exhaustive list of inclusion and exclusion criteria can be found at ClinicalTrials.gov (NCT03311477).^29^

### 2.3 Assessments

Safety assessments included treatment‐emergent adverse events (TEAEs) graded according to the National Cancer Institute Common Terminology Criteria for Adverse Events version 4.03. TEAEs, vital signs, physical examinations, electrocardiograms, laboratory tests, and ECOG performance status were monitored during the study. Any grade ≥3 study drug‐related nonhematologic toxicity was documented as a DLT, except as follows: grade 3 nausea or vomiting lasting ≤48 hours that was successfully managed with antiemetics; grade 3 diarrhea successfully managed with antidiarrheal; grade 3 constipation lasting ≤7 days; grade 3 acute infusion reaction that resolved to grade ≤1 within 24 hours after the end of dosing; grade 3 and 4 laboratory tests including but not limited to lactate dehydrogenase, alkaline phosphatase, and gamma‐glutamyl transferase that were considered nonclinically significant by the investigator; liver function test abnormalities; grade 3 and 4 electrolyte imbalance unless associated with clinical symptomatology despite supplementation; and grade 3 hyperglycemia that was manageable without hospitalization. The following hematologic toxicities were documented as a DLT: any grade 4 hematologic toxicity (excluding febrile neutropenia or leukopenia lasting for ≤7 days, or lymphopenia); anemia requiring red blood cell transfusion; thrombocytopenia requiring platelet transfusion; grade 3 thrombocytopenia in the presence of grade ≥2 bleeding; and grade ≥3 febrile neutropenia. Toxicity management criteria are described in the Supplementary appendix.

Serial PK samples were collected for teliso‐v and MMAE in cycle 1 and cycle 3 predose and approximately 30 minutes after the end of infusion on day 1, and days 2, 4, 8, and 15 at the scheduled visits. PK samples were also collected on cycles 2 and 4 predose and approximately 30 minutes after the end of infusion on day 1. PK parameters such as peak concentration (C_max_), time to peak concentration, area under the concentration‐time curve (AUC), and terminal half‐life (t_1/2_) were determined using noncompartmental methods.

Computed tomography and/or magnetic resonance imaging were performed at screening, and every two cycles after initiation of teliso‐v. Evaluation of tumor response to determine the objective response rate (ORR), progression‐free survival (PFS), and duration of response was based on RECIST version 1.1 criteria.

IHC was retrospectively performed (by central laboratory analysis) on archival tissue biopsies to assess c‐Met protein expression utilizing the SP44 antibody. The H‐score was calculated by utilizing the formula: 3 × percentage of strongly staining cells +2 × percentage of moderately staining cells +1 × percentage of weakly staining nuclei.

### 2.4 Statistical analysis

All patients who received at least 1 dose of teliso‐v were included in the safety assessment. All data were summarized and tabulated by dose cohort. Categoric data were summarized in terms of frequency counts and percentages. Continuous data were summarized using descriptive statistics, including number of observations, mean, standard deviation, median, minimum, and maximum. No formal statistical analysis was performed for efficacy variables, which were all exploratory in nature. ORR was defined as the proportion of patients with a confirmed partial response (PR) or complete response (CR). Duration of response was defined as time from a patient's initial objective response to the first date of disease progression (PD) or death. The overall disease control rate was defined as the proportion of patients with CR, PR, or stable disease (SD). PFS was defined as time from the first day of teliso‐v treatment to disease progression or death or up to 24 months for those who remained on treatment. PFS was estimated using the Kaplan–Meier method. Efficacy analyses are presented with two‐sided exact 95% binomial CI.

## 3 RESULTS

### 3.1 Patient demographics and baseline characteristics

Patients were enrolled from November 2017 to April 2018. Database with collected study data was locked in August 2019. In total, nine patients with solid tumors, including pancreatic cancer, ovarian cancer, urothelial carcinoma, thymic cancer, esophageal cancer, breast cancer, liposarcoma (n = 1 each), and NSCLC (n = 2) were enrolled and received at least one dose of teliso‐v. All nine patients had metastatic disease at the time of enrollment, and median time from initial diagnosis to study entry was 48.2 months (range 17.0–156.3). Demographics and baseline characteristics of the study population are summarized in Table 1. Median age was 58 years (range 44–74) and all patients had ECOG performance status 0 or 1. Three patients were enrolled at the 2.4‐mg/kg dose level and six patients received dose escalation at the 2.7‐mg/kg dose level. Patients were heavily pretreated and did not achieve a CR on prior therapies. c‐Met protein overexpression was retrospectively analyzed in all patients, and none of the enrolled patients had c‐Met IHC scores above the threshold utilized in the global phase 1 study. Median treatment duration with teliso‐v was 19.1 weeks (range 3.1–47.6) in the overall population, 15.0 weeks (range 9.1–20.9) in the 2.4‐mg/kg cohort (n = 3), and 30.6 weeks (range 3.1–47.6) for the 2.7‐mg/kg cohort (n = 6). All nine patients discontinued teliso‐v; primary reasons for discontinuation were radiologic progression (n = 6; 67%) and withdrawal of consent (n = 3, 33%).

**TABLE 1:** Patient demographics and baseline characteristics

Characteristic	Teliso‐v 2.4 mg/kg (*n* = 3)	Teliso‐v 2.7 mg/kg (*n* = 6)	Total (*N* = 9)
Median age, years (range)	55 (44–73)	58.5 (44–74)	58 (44–74)
Gender, *n* (%)			
Female	1 (33)	3 (50)	4 (44)
Male	2 (67)	3 (50)	5 (56)
Primary cancer types, *n* (%)			
Breast	1 (33)	0	1 (11)
Esophageal	1 (33)	0	1 (11)
Liposarcoma	0	1 (17)	1 (11)
Lung (non‐small cell)	1 (33)	1 (17)	2 (22)
Ovarian	0	1 (17)	1 (11)
Pancreatic	0	1 (17)	1 (11)
Thymus	0	1 (17)	1 (11)
Urothelial	0	1 (17)	1 (11)
ECOG PS, *n* (%)			
0	2 (67)	0	2 (22)
1	1 (33)	6 (100)	7 (78)
2	0	0	0
Number of prior therapies, *n* (%)			
1	0	0	0
2	0	2 (33)	2 (22)
≥3	3 (100)	4 (67)	7 (78)
Best response to prior therapy, *n* (%)			
Complete response	0	0	0
Partial response	2 (67)	5 (83)	7 (78)
Stable disease	0	1 (17)	1 (11)
Progressive disease	1 (33)	0	1 (11)
c‐Met H‐score, *n* (%)			
Cytoplasm, ≤20	3 (100)	6 (100)	9 (100)
Membrane, ≤50	3 (100)	6 (100)	9 (100)

Abbreviation: ECOG, Eastern Cooperative Oncology Group; PS, performance status; teliso‐v, telisotuzumab vedotin.

### 3.2 Safety

During dose escalation, no DLTs were reported for either dose level; therefore, the RP2D was established at 2.7 mg/kg Q3 W.

All nine patients experienced at least one TEAE and at least one TEAE related to teliso‐v. Most frequent TEAEs (>20% of total patients) related to teliso‐v are summarized in Table 2; peripheral sensory neuropathy (44%), and nausea, decreased appetite, and decreased white blood cell count (33% each) were the most common. TEAEs, regardless of relationship to teliso‐v, reported for three or more patients (≥33%) were peripheral sensory neuropathy (44%), and decreased appetite, nausea, decreased weight, and decreased white blood cell count (33% each) (Table S1). Grade ≥3 TEAEs, regardless of causality to teliso‐v, were decreased neutrophil count and hypoalbuminemia in two patients (22%) each, and hypophosphatemia and fatigue in one patient (11%) each. The only grade 4 TEAE was decreased neutrophil count, reported for one patient (11%; 2.7‐mg/kg dose level). Three serious AEs occurred in a total of two patients. One patient experienced grade 3 fatigue and grade 2 decreased appetite, and the other patient experienced grade 2 gait disturbance. From the three serious AEs, only the grade 2 event of gait disturbance was considered teliso‐v related (2.7‐mg/kg dose level). This event was the only TEAE leading to treatment discontinuation. AEs led to withdrawal of consent for three patients: generalized muscle weakness (grade 2, n = 1), peripheral sensory neuropathy (grade 2, n = 1), right retinal pigment epithelial detachment (grade 1, n = 1). No deaths were reported for either dose level.

**TABLE 2:** Summary of treatment‐emergent adverse events related to teliso‐v

	Teliso‐v 2.4 mg/kg (*n* = 3)	Teliso‐v 2.7 mg/kg (*n* = 6)	Total (*N* = 9)
TEAEs related to teliso‐v [>20% of total patients], *n* (%)^†^			
Peripheral sensory neuropathy	1 (33)	3 (50)	4 (44)
Nausea	1 (33)	2 (33)	3 (33)
Decreased appetite	2 (67)	1 (17)	3 (33)
White blood cell count decreased	0	3 (50)	3 (33)
Fatigue	1 (33)	1 (17)	2 (22)
Malaise	2 (67)	0	2 (22)
Alanine aminotransferase increased	0	2 (33)	2 (22)
Aspartate aminotransferase increased	0	2 (33)	2 (22)
Neutrophil count decreased	0	2 (33)	2 (22)
Weight decreased	1 (33)	1 (17)	2 (22)
Punctate keratitis	1 (33)	1 (17)	2 (22)
Peripheral neuropathy	2 (67)	0	2 (22)
Alopecia	0	2 (33)	2 (22)

Abbreviation: TEAE, treatment‐emergent adverse event; teliso‐v, telisotuzumab vedotin.

† As assessed by the investigator.

AEs of special interest (AESIs) were also assessed, including bone marrow suppression, peripheral neuropathy, edema, decreased testosterone, and ocular events. Five of nine (56%) patients experienced hematologic AEs, including one (33%) patient at the 2.4‐mg/kg dose level and four (67%) patients at the 2.7‐mg/kg dose level. Hematologic AEs identified in one or more patients were: decreased white blood cell count (n = 3, grades 1 and 2) and decreased neutrophil count (n = 2, grades 2, 3, and 4); all occurred at the 2.7‐mg/kg dose level with multiple events experienced over the course of treatment.. All hematologic AEs were considered to be teliso‐v related, and none were considered serious. Six of nine (67%) patients experienced peripheral neuropathy events (grades 1 and 2), including three patients each at the 2.4‐ and 2.7‐mg/kg dose levels. These events were considered to be treatment related, and three events resulted in interruption of teliso‐v. Hypoalbuminemia (grade 2 and 3) was experienced by two (22%) of the nine patients (both treated with the 2.7‐mg/kg dose), with one (11%) of those patients also experiencing peripheral edema. The peripheral edema event and one of the hypoalbuminemia events were considered related to study drug. Two (22%) patients, both at the 2.4‐mg/kg dose level, reported TEAEs of blood testosterone decreased (grade 1) and muscular weakness (grade 2), which were considered related to teliso‐v treatment. Four (44%) patients, including three patients treated at the 2.7‐mg/kg dose level, reported TEAEs that were identified as ocular AESIs. These included punctate keratitis (two patients), dry eye (one patient), keratitis (one patient), and visual impairment (one patient). These ocular events were grade 1 or 2 in severity and considered related to treatment.

### 3.3 Pharmacokinetics

**FIGURE 1:**
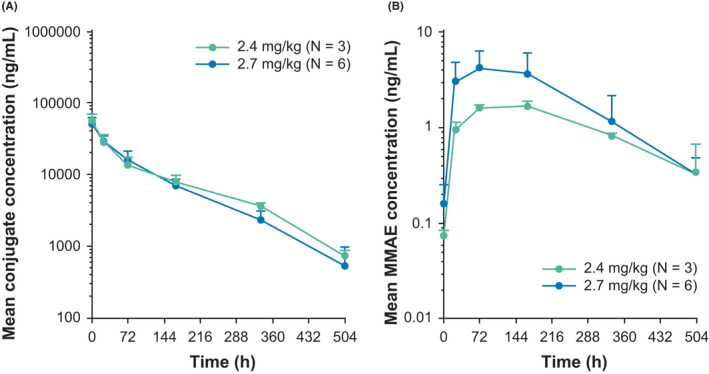
Preliminary PK profiles for (A) teliso‐v and (B) MMAE. PK profiles (mean +standard deviation) after IV infusion (2.4 mg/kg and 2.7 mg/kg) in cycle 1 on a Q3 W dosage schedule. Log‐linear scales. IV, intravenous; MMAE, monomethyl auristatin E; PK, pharmacokinetic; Q3 W, every 3 weeks; teliso‐v, telisotuzumab vedotin

The systemic exposures of teliso‐v were approximately dose proportional across both dose level cohorts in cycle 1 (Figure 1). Following the first administration of teliso‐v in cycle 1, the preliminary geometric mean (percentage coefficient of variation [%CV]) of the serum C_max_ of teliso‐v was 55.5–63.0 µg/ml (%CV: 22%–26%), and the AUC from zero to infinity was approximately 4,400–5,200 µg*h/ml (%CV: 19%–23%) (Table S2). The preliminary geometric mean (%CV) plasma C_max_ for MMAE following the first dose in cycle 1 was 1.69–2.80 ng/ml (%CV: 10%–77%) (Table S2). The t_1/2_ for teliso‐v and the MMAE payload were determined to be approximately 3–4 days and 4–6 days, respectively (N = 9), and the accumulation for teliso‐v and MMAE in cycle 3 following multiple dosing Q3 W was minimal (approximately 1.1–1.2 fold).

### 3.4 Antitumor activity

**FIGURE 2:**
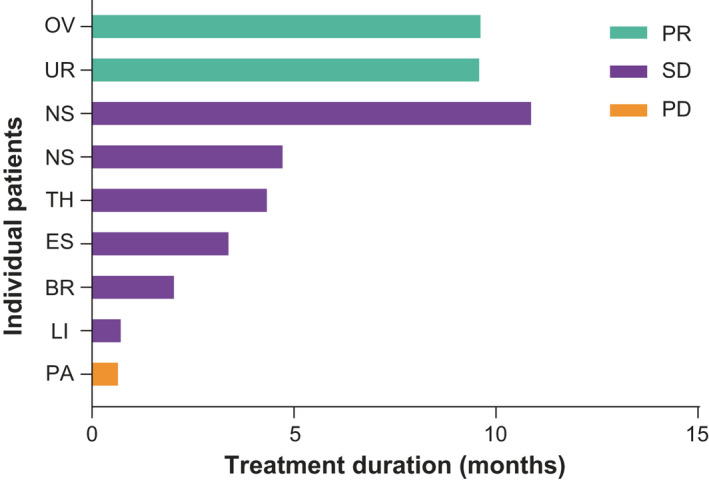
Best overall response per patient and duration of treatment. Duration of treatment with overall responses is shown in different types of solid tumors. Two patients had PR, six patients had SD, and one patient had PD. BR, breast cancer; ES, esophageal cancer; LI, liposarcoma; NS, non‐small cell lung cancer; OV, ovarian cancer; PA, pancreatic cancer; PD, progressive disease; PR, partial response; SD, stable disease; TH, thymic cancer; UR, urothelial carcinoma

Figure 2 illustrates the best response achieved by each patient and the duration of teliso‐v treatment. Two patients (one with urothelial cancer and the other with ovarian cancer) treated at the 2.7‐mg/kg dose level achieved PRs, after an average of 9.6 months of treatment. The c‐Met status on archival tissue samples from these patients was below the H‐score cut‐off utilized in the teliso‐v phase 1 study conducted outside of Japan^3^ (c‐Met membrane H‐score of 10 and 35 respectively). The patient with urothelial and metastatic lung disease achieved a PR after cycle 2 (Figure S1). Another patient with ovarian cancer and abdominal lymph node metastatic disease achieved a PR after cycle 4. There were six patients with SD (NSCLC [n =2], thymic cancer, esophageal cancer, breast cancer, liposarcoma [n =1, each]) treated for 0.8–10.9 months. The ORR was 22% (95% CI: 2.8–60.0) and the overall disease control rate was 89% (95% CI: 51.8–99.7) (Table S3). Median duration of response was 8.2 months (95% CI: 7.2–9.1) and median PFS was 7.1 months (95% CI: 1.2–10.4). Four patients (thymic cancer, NSCLC, ovarian cancer, and urothelial cancer) showed a reduction in tumor size from baseline (Figure S2).

## 4 DISCUSSION

Dysregulated c‐Met activity has been associated with oncogenic potential and has emerged as an effective prognostic biomarker in solid tumors.^30^ Several new compounds that target the c‐Met pathway, such as kinase inhibitors, have recently gained approval. Among these are tepotinib and capmatinib, both of which have been evaluated in phase 2 studies of patients with *MET* exon 14‐mutated NSCLC.^25^, ^26^ In the tepotinib study, the primary endpoint of ORR by independent review committee was reached by 51.4% of evaluable patients.^25^ In a multicohort phase 2 study, capmatinib achieved ORR rates of 39.1% in those treated with one to two prior lines of treatment, and 71.4% in treatment‐naive patients.^26^ However, by targeting only the subpopulation of patients with *MET* exon 14 mutations,^25^, ^26^, ^27^, ^28^, ^30^, ^31^ or in the case of molecules that target only *MET* gene amplification with high *MET* copy number,^32^ these spectrum‐selective c‐Met inhibitors have limited therapeutic scope. In NSCLC, for instance, *MET* genomic alterations, such as *MET* gene amplification or *MET* exon 14 mutations, are present in <5% of patients,^13^, ^14^ whereas 30%–50% of patients have tumors that overexpress c‐Met in the absence of *MET* genomic alterations.^33^ As a consequence, most patients whose tumors demonstrate aberrant c‐Met protein overexpression are potentially nonresponsive to the c‐Met–targeted therapies currently under development. Of note, the classification of c‐Met protein overexpression depends on the IHC cut‐off, which is defined for the specific antibody and assay being utilized. In the case of teliso‐v, the IHC cut‐off with the SP44 antibody was defined as an H‐score ≥150. None of the retrospectively analyzed samples from patients in this study met this threshold for c‐Met overexpression. This may indicate that the prevalence of c‐Met overexpression varies among tumor types.

Teliso‐v, a potent ADC targeted to c‐Met, was designed to be effective in tumors with c‐Met protein overexpression, irrespective of *MET* gene amplification. In a global phase 1 study of teliso‐v in patients with advanced solid tumors (NCT02099058), three of 14 patients with c‐Met‐positive NSCLC had a PR; none of these responders had *MET* genomic alterations.^3^ Further, teliso‐v was well tolerated at a dose of 2.7 mg/kg Q3 W.^3^ Nevertheless, this global study did not include Japanese patients, thus providing the rationale for the present phase 1 study, which evaluated the safety, tolerability, and PK of teliso‐v in Japanese patients with advanced solid tumors. The safety and PK of teliso‐v were assessed according to a 3 + 3 dose‐escalation design at a dose level of either 2.4 or 2.7 mg/kg Q3 W. Nine patients with eight different tumor types were enrolled, and all patients had metastatic disease at the time of enrollment.

TEAEs were overall manageable, with no DLTs observed and safety findings similar to the global phase 1 study.^3^ In clinical studies evaluating ADCs that utilize the MMAE warhead, hematologic toxicities related to peripheral neuropathy and bone marrow suppression (neutropenia, anemia, thrombocytopenia) have been commonly reported. In the present study, TEAEs/AESIs that were possibly related to MMAE occurred at a similar grade and frequency as the global teliso‐v study.^3^ Teliso‐v, by virtue of targeting c‐Met, has also displayed toxicities possibly related to its on‐target action, including peripheral edema, hypoalbuminemia, and decreased testosterone. In addition, ocular toxicities such as keratitis that were likely to be related to teliso‐v were observed more frequently in Japanese patients compared with the non‐Japanese population. The frequency of corneal epithelium disorder was 44.4% (four of nine patients) in Japanese patients and 7.8% (nine of 116 patients) in non‐Japanese patients (data on file) treated with teliso‐v monotherapy. However, the sample size in the present study was limited, and the occurrence of toxicities requires further investigation. The ocular toxicities were generally low‐grade, manageable, and reversible.

As with safety, the PK parameters of teliso‐v in Japanese patients were similar to those observed in non‐Japanese patients,^3^ suggesting no remarkable ethnic difference in PK. This was also the case for another ADC, brentuximab vedotin, which links MMAE to a CD30‐specific antibody. In a phase 1/2 study of Japanese patients with CD30‐positive Hodgkin lymphoma or systemic anaplastic large cell lymphoma, the safety profile of brentuximab vedotin was similar for US and Japanese patients, and no remarkable ethnic differences in PK were found.^34^ In the present study, the maximum tolerated dose was not reached on the basis of the study design, and the RP2D of teliso‐v as a single agent in Japanese patients with advanced solid tumors is 2.7 mg/kg Q3 W, confirming the RP2D determined in the phase 1 study outside of Japan.

In the efficacy analysis, two of nine patients achieved a PR, including one patient with ovarian cancer and one patient with urothelial cancer. Both patients had a c‐Met H‐score below 150, the score defined as the IHC cutoff in the teliso‐v phase 1 study conducted outside of Japan.^3^ In that study, all patients with a PR had c‐Met overexpression.^3^ This apparent difference in c‐Met expression and association with response is potentially reconciled on the basis of the use of archival tumor tissue for c‐Met evaluation in the present study. In addition, the tumor tissue of the patient with ovarian cancer showed heterogeneous expression of c‐Met (data not shown). Intratumoral biomarker heterogeneity may affect any correlation between efficacy and biomarker score, and c‐Met protein expression level may change over time or as a result of prior treatment. In addition to the use of archival tumor tissue, this study was further limited by the small number of patients and absence of patient selection on the basis of c‐Met overexpression, which potentially limits the accurate assessment of teliso‐v antitumor efficacy.

In conclusion, teliso‐v was well‐tolerated at a dose of 2.7 mg/kg Q3 W in Japanese patients with advanced solid tumors. The overall risk/benefit of teliso‐v in Japanese patients supports continued investigation of teliso‐v for the treatment of solid tumors. Further investigation of teliso‐v in Japanese patients with cancer is warranted, on the basis of the reported response and tolerability in this study. A global phase 2 trial evaluating teliso‐v in patients with NSCLC, including patients from Japan, is ongoing (NCT03539536).

## AUTHOR CONTRIBUTION

**Yutaka Fujiwara:** Grants from AbbVie, during the conduct of the study; grants and personal fees from AstraZeneca, BMS, MSD, Novartis; grants from Chugai, Daiichi Sankyo, Eisai, Eli Lilly, Incyte, Merck Serono; personal fees from Ono, outside the submitted work. **Noboru Yamamoto:** Grants from Astellas, Chugai, Eisai, Taiho, BMS, Pfizer, Novartis, Eli Lilly, AbbVie, Daiichi Sankyo, Bayer, Boehringer Ingelheim, Kyowa Hakko Kirin, Takeda, Ono, Janssen Pharma, MSD, Merck, GSK; personal fees from BMS, Pfizer, AstraZeneca, Eli Lilly, Ono, Chugai, Sysmex. **Hirotsugu Kenmotsu:** Grants and personal fees from AstraZeneca K.K., grants and personal fees from Chugai Pharmaceutical Co, Ltd., personal fees from Ono Pharmaceutical Co, Ltd., grants and personal fees from Boehringer Ingelheim, personal fees from Eli Lilly K.K, personal fees from Kyowa Hakko Kirin Co., Ltd., personal fees from Bristol‐Myers Squibb, personal fees from MSD, personal fees from Novartis Pharma K.K., grants from Daiichi‐Sankyo Co., Ltd., personal fees from Pfizer K.K. **Kan Yonemori:** Honoraria from Eisai, Pfizer, AstraZeneca; advisor to Eisai, Chugai, Ono, Takeda, Novartis. **Toshio Shimizu:** Grants from Novartis, Eli Lilly, Daiichi‐Sankyo, Eisai, Bristol‐Myers Squibb, Takeda Oncology, Incyte, Astellas, Chordia Therapeutics, 3D‐Medicine, Symbio Pharmaceuticals, PharmaMar, Five Prime, AstraZeneca, and AbbVie; Principal investigator (ABBV‐151, ABBV‐184, ABBV‐368, ABBV‐927); Honoraria (Regular Member of IRB Scientific Review) from the Consortium on Harmonization of Institutional Requirements for Clinical Research (CHAIR) Joint Scientific Committee Review Member for Phase 1 trials in Hong Kong, HKSAR China. **Christopher Ocampo, Apurvasena Parikh, Sumiko Okubo, and Kazuteru Fukasawa:** AbbVie employees and may own stock. **Haruyasu Murakami:** Grants from AbbVie, AstraZeneca, Astellas, Taiho Pharmaceutical, Lilly Japan, Takeda, Roche Daiichi Sankyo; personal fees from AstraZeneca, Chugai Pharma, Lilly Japan, Ono Pharmaceutical, Bristol‐Myers Squibb Japan, Merck Sharp & Dohme, Taiho Pharmaceutical, Pfizer, Novartis, Boehringer Ingelheim.

## Supporting information

Figure S1

Figure S2

Table S1

Table S2

Table S3

Supplementary Material

## Data Availability

AbbVie is committed to responsible data sharing regarding the clinical trials we sponsor. This includes access to anonymized, individual and trial‐level data (analysis data sets), as well as other information (eg, protocols and Clinical Study Reports), as long as the trials are not part of an ongoing or planned regulatory submission. This includes requests for clinical trial data for unlicensed products and indications. These clinical trial data can be requested by any qualified researchers who engage in rigorous, independent scientific research, and will be provided following review and approval of a research proposal and Statistical Analysis Plan (SAP) and execution of a Data Sharing Agreement (DSA). Data requests can be submitted at any time and the data will be accessible for 12 months, with possible extensions considered. For more information on the process, or to submit a request, visit the following link: https://www.abbvie.com/our‐science/clinical‐trials/clinical‐trials‐data‐and‐information‐sharing/data‐and‐information‐sharing‐with‐qualified‐researchers.html
