# Supplementary material for: Phase 1 study of telisotuzumab vedotin in Japanese patients with advanced solid tumors
Source: Cancer Med. 2021 Mar 6;10(7):2350–8. doi: 10.1002/cam4.3815 (PMC7982615; doi:10.1002/cam4.3815)
Supplement: Supplementary file 1 — Figure S1 [file CAM4-10-2350-s001.docx]

**SUPPORTING INFORMATION**

**Supplementary figure**

**Figure S1.** Computed tomography images of the lung metastasis of a patient with non-small cell lung cancer at baseline and after 27 weeks of teliso-v treatment. Arrows indicate the location of the tumor. (A) Tumor size at baseline and (B) tumor size after 27 weeks. The sum of the tumor size at baseline was 64.4 mm, which was then reduced to 17.3 mm (73% reduction) at 27 weeks after teliso-v initiation. teliso-v, telisotuzumab vedotin.

**
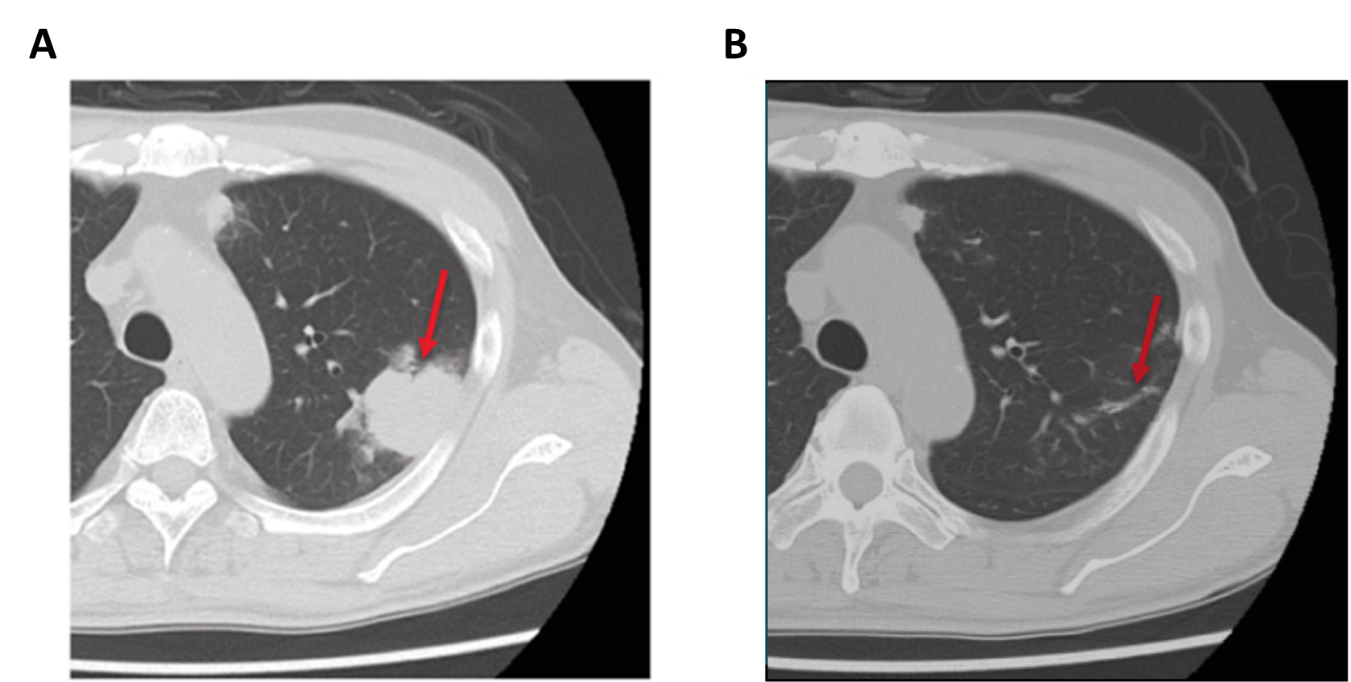
**
