# Supplementary material for: Phase 1 study of telisotuzumab vedotin in Japanese patients with advanced solid tumors
Source: Cancer Med. 2021 Mar 6;10(7):2350–8. doi: 10.1002/cam4.3815 (PMC7982615; doi:10.1002/cam4.3815)

**SUPPORTING INFORMATION**

**Supplementary figure**

**Figure S2.** Best percentage change in size of target lesions from baseline in all patients with ≥1 postbaseline tumor assessment. Change in size of target lesions, expressed as percentage from baseline, is shown for different types of solid tumors. BR, breast cancer; ES, esophageal cancer; LI, liposarcoma; NS, non-small cell lung cancer; OV, ovarian cancer; PA, pancreatic cancer; TH, thymic cancer; UR, urothelial carcinoma.


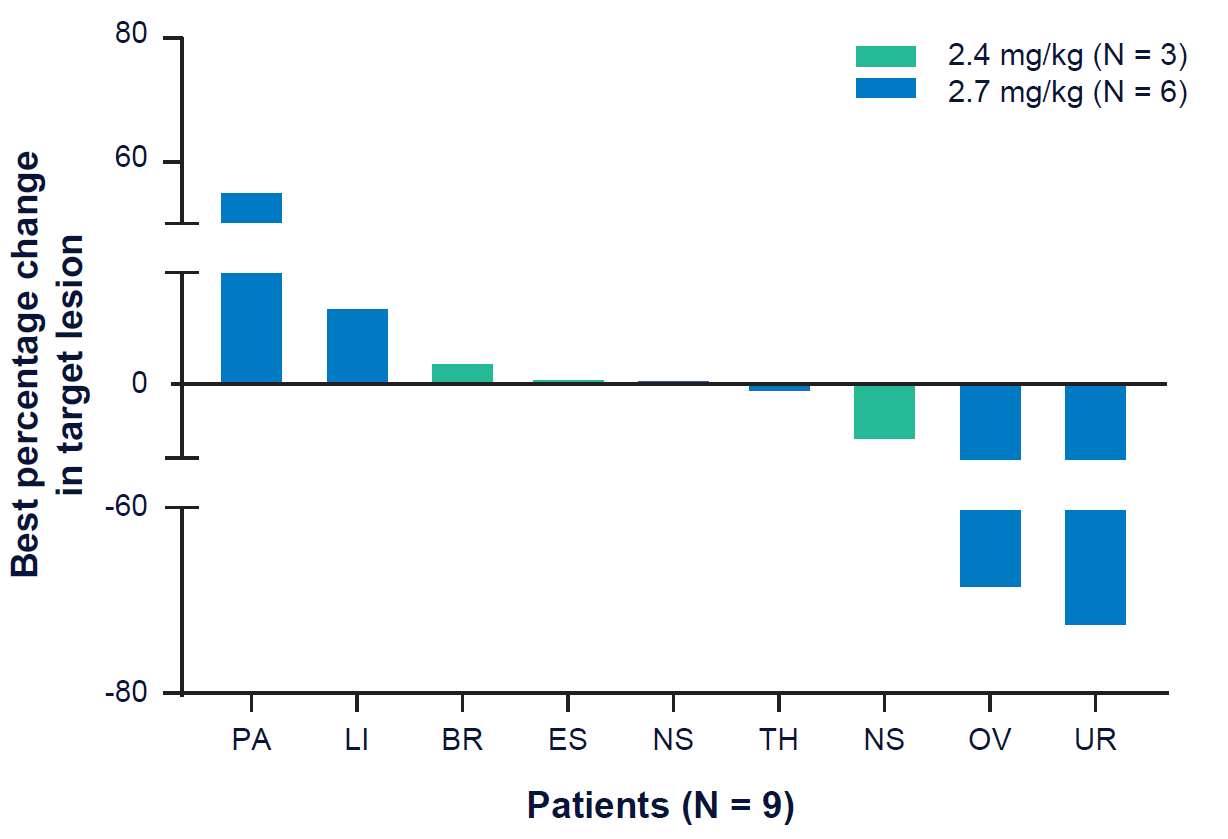

Supplement: Supplementary file 2 — Figure S2 [file CAM4-10-2350-s004.docx]
