# Supplementary material for: Phase 1 study of telisotuzumab vedotin in Japanese patients with advanced solid tumors
Source: Cancer Med. 2021 Mar 6;10(7):2350–8. doi: 10.1002/cam4.3815 (PMC7982615; doi:10.1002/cam4.3815)
Supplement: Supplementary file 3 — Table S1 [file CAM4-10-2350-s003.docx]

**SUPPORTING INFORMATION**

**Table S1. Summary of treatment-emergent adverse events** **regardless of relationship to teliso-v**

|  | **Teliso-v**  **2.4 mg/kg**  **(n = 3)** | **Teliso-v**  **2.7 mg/kg**  **(n = 6)** | **Total**  **(N = 9)** |
| --- | --- | --- | --- |
| **TEAEs** **overall [>20% total patients], n (%)** |  | | |
| Peripheral sensory neuropathy | 1 (33) | 3 (50) | 4 (44) |
| Decreased appetite | 2 (67) | 1 (17) | 3 (33) |
| Nausea | 1 (33) | 2 (33) | 3 (33) |
| Weight decreased | 1 (33) | 2 (33) | 3 (33) |
| White blood cell count decreased | 0 | 3 (50) | 3 (33) |
| Alanine aminotransferase increased | 0 | 2 (33) | 2 (22) |
| Alopecia | 0 | 2 (33) | 2 (22) |
| Aspartate aminotransferase increased | 0 | 2 (33) | 2 (22) |
| Back pain | 1 (33) | 1 (17) | 2 (22) |
| Constipation | 0 | 2 (33) | 2 (22) |
| Diarrhea | 1 (33) | 1 (17) | 2 (22) |
| Fatigue | 1 (33) | 1 (17) | 2 (22) |
| Anemia | 1 (33) | 1 (17) | 2 (22) |
| Hypoalbuminemia | 0 | 2 (33) | 2 (22) |
| Malaise | 2 (67) | 0 | 2 (22) |
| Peripheral neuropathy | 2 (67) | 0 | 2 (22) |
| Neutrophil count decreased | 0 | 2 (33) | 2 (22) |
| Punctate keratitis | 1 (33) | 1 (17) | 2 (22) |
| TEAE, treatment-emergent adverse event; teliso-v, telisotuzumab vedotin. | | | |
