# Supplementary material for: Phase 1 study of telisotuzumab vedotin in Japanese patients with advanced solid tumors
Source: Cancer Med. 2021 Mar 6;10(7):2350–8. doi: 10.1002/cam4.3815 (PMC7982615; doi:10.1002/cam4.3815)
Supplement: Supplementary file 4 — Table S2 [file CAM4-10-2350-s005.docx]

**SUPPORTING INFORMATION**

**Table S2. Summary of teliso-v pharmacokinetics**

| **Geometric mean (%CV) in cycle 1** | **Teliso-v**  **2.4 mg/kg (n = 3)** | **Teliso-v**  **2.7 mg/kg (n = 6)** |
| --- | --- | --- |
| Conjugate | | |
| T_max_,^a^ h | 1 (1–1) | 1 (1–1) |
| C_max_, µg/mL | 55.5 (26) | 63.0 (22) |
| AUC_inf_, µg*h/mL | 4437 (19) | 5202 (23) |
| t_1/2_,^b^ days | 4.18 (3.8–4.7) | 2.91 (2.5–3.9) |
| MMAE | | |
| T_max_,^a^ h | 168.5 (72.5–168.5) | 168.5 (72.5–168.5) |
| C_max_, ng/mL | 1.69 (10) | 2.80 (77) |
| AUC_tau_, ng*h/mL | 535 (7) | 764 (78) |
| t_1/2_^b^, days | 5.80 (4.5–7.8) | 4.01 (3–5.6)^c^ |
| ^a^T_max_ presented as median (min-max); ^b^t_1/2_ presented as harmonic mean (min-max); ^c^N = 4.  %CV, percentage coefficient of variation; AUC_inf_, area under the serum concentration-time curve from zero to infinity; AUC_tau_, area under the serum concentration-time curve within the dosing interval; C_max_, peak concentration; MMAE, monomethyl auristatin E; t_1/2_, terminal half-life; teliso-v, telisotuzumab vedotin; T_max_, time to peak concentration. | | |
