# Supplementary material for: Phase 1 study of telisotuzumab vedotin in Japanese patients with advanced solid tumors
Source: Cancer Med. 2021 Mar 6;10(7):2350–8. doi: 10.1002/cam4.3815 (PMC7982615; doi:10.1002/cam4.3815)
Supplement: Supplementary file 5 — Table S3 [file CAM4-10-2350-s002.docx]

**SUPPORTING INFORMATION**

**Table S3. Efficacy outcomes**

|  | **Teliso-v**  **2.4 mg/kg**  **(n = 3)** | **Teliso-v**  **2.7 mg/kg**  **(n = 6)** | **Total**  **(N = 9)** |
| --- | --- | --- | --- |
| Objective response rate, n (%) | 0 | 2 (33) | 2 (22) |
| Complete response | 0 | 0 | 0 |
| Partial response | 0 | 2 (33) | 2 (22) |
| Stable disease, n (%) | 3 (100) | 3 (50) | 6 (67) |
| Progressive disease, n (%) | 0 | 1 (17) | 1 (11) |
| Disease control rate, n (%)  [95% CI] | 3 (100)  [NA] | 5 (83)  [35.9–99.6] | 8 (89)  [51.8–99.7] |
| Median duration of overall response, months  [95% CI] | – | 8.2  [7.2–9.1] | 8.2  [7.2–9.1] |
| Median PFS, months [95% CI] | 4.1 [2.8–7.1] | 10.4 [1.2–12.0] | 7.1 [1.2–10.4] |
| PFS estimate at 6 months, % [95% CI] | 33.3 [0.9–77.4] | 83.3 [27.3–97.5] | 64.0 [23.8–86.6] |
| PFS estimate at 9 months, % [95% CI] | – | 83.3 [27.3–97.5] | 48.0 [12.3–76.9] |
| NA, not applicable; PFS, progression free survival; teliso-v, telisotuzumab vedotin. | | | |
