# Supplementary material for: Phase 1 study of telisotuzumab vedotin in Japanese patients with advanced solid tumors
Source: Cancer Med. 2021 Mar 6;10(7):2350–8. doi: 10.1002/cam4.3815 (PMC7982615; doi:10.1002/cam4.3815)
Supplement: Supplementary file 6 — Supplementary Material [file CAM4-10-2350-s006.docx]

**Supplementary appendix**

*Toxicity management criteria*

For patients who received the first dose of teliso-v and were clinically stable but experienced a reversible toxicity, the dose of study drug could be delayed for up to 21 days (or up to 42 days for peripheral neuropathy, hypoalbuminemia, or peripheral edema) after the scheduled dosing date. Depending upon the nature and rapidity of the reversibility of the toxicity, the patient could continue the same dose level or reduce the dose by 0.3 mg/kg at the investigator’s discretion (larger dose reductions were allowed after consultation with the sponsor’s [AbbVie, North Chicago, IL] Medical Monitor). Up to three dose reductions were allowed to manage toxicity, after which the patient would be discontinued from therapy. Patients who experienced an adverse event that led to a delay in teliso-v dosing for >21 days (or >42 days for neuropathy, hypoalbuminemia, or edema) from the scheduled dose were discontinued from study.

While the discretion of the investigator should be used for patient management with regard to toxicities (eg, acute infusion reactions, neuropathy and bone marrow suppression, hypoalbuminemia and edema, hepatotoxicity, decreased testosterone, pneumonitis), suggested guidelines are provided in the study protocol.
